# Impact of Diabetes Mellitus On In-Hospital Mortality of COVID-19 Patients in Japan Since COVID-19 Became a Common Infectious Disease

**DOI:** 10.7759/cureus.66373

**Published:** 2024-08-07

**Authors:** Yohei Fujita, Masahiro Hatazaki, Satoshi Fujimi

**Affiliations:** 1 Department of Diabetes and Endocrinology, Osaka General Medical Center, Osaka, JPN; 2 Division of Trauma and Surgical Critical Care, Osaka General Medical Center, Osaka, JPN

**Keywords:** omicron variant, japanese, mortality, diabetes mellitus, coronavirus disease 2019 (covid-19)

## Abstract

Aim: The number of severe cases of coronavirus disease 2019 (COVID-19) has been decreasing since the emergence of the Omicron variant at the end of 2021. COVID-19 has become a common infectious disease in Japan and was downgraded to a category five infectious disease on May 8, 2023. This study aimed to compare the impact of diabetes mellitus on in-hospital mortality in COVID-19 patients since COVID-19 became a common infectious disease.

Patients and methods: We conducted a retrospective observational study using data from an advanced critical care center in Osaka, Japan. The study included 1,381 patients of COVID-19 admitted to the center between March 1, 2020, and May 7, 2023, before COVID-19 became a category five infectious disease in Japan. Individuals younger than 18 years and pregnant women were excluded. We divided the patients into two groups: pre- and post-Omicron epidemic groups. The primary endpoint of the study was the in-hospital mortality, and the prognostic impact of diabetes mellitus was compared between the groups.

Results: The Kaplan-Meier curve showed a significantly lower rate of in-hospital mortality in the post-Omicron epidemic group than in the pre-Omicron epidemic group. The hazard ratio (HR) was 1.83 (95% CI, 1.36-2.50; p < 0.0001). Patients with diabetes mellitus had higher in-hospital mortality in both the pre- and post-Omicron epidemic groups; their HRs were 1.39 (95% CI, 1.21-1.59; p < 0.0001) and 1.45 (95% CI, 1.15-1.83; p = 0.0012), respectively. Diabetes mellitus had no significant interaction effect on the association between the post-Omicron epidemic and in-hospital mortality (p for interaction = 0.2154).

Conclusion: Diabetes mellitus may continue contributing to COVID-19 in-hospital mortality in the future, as the Omicron sub-strain may still be prevalent.

## Introduction

Severe acute respiratory syndrome coronavirus 2 (SARS-CoV-2), the causative virus of novel coronavirus disease 2019 (COVID-19), belongs to the genus Betacoronavirus of the Coronaviridae family and is an enveloped virus with a single-stranded and plus-stranded RNA genome consisting of approximately 30,000 bases [1]. The virus was first identified in Wuhan, Hubei Province, China, in December 2019 [2], after which COVID-19 became a global pandemic [3,4]. In March 2020, our hospital began inpatient care for severe COVID-19 patients.

The SARS-CoV-2 mutant strains that emerged at the end of 2020 have genetic variants that affect infectivity, transmissibility, virulence (severity of infection by the pathogen), and antigenicity. The B.1.1.7 strain (Alpha), B.1.617.2 strain (Delta), and B.1.1.529 strain (Omicron) interchangeably formed the COVID-19 epidemic [5,6]. Since the outbreak of Omicron at the end of 2021, numerous sub-strains of Omicron have occurred [7]. Omicron and its sub-strain are much more infectious and transmissible than the pre-Omicron strains, but their virulence has decreased, and the proportion of severe cases has declined [5,8]. On May 4, 2023, the World Health Organization (WHO) declared an end to the public health emergency of international concern. Japan's Infectious Diseases Act classifies infectious diseases from one to five categories, considering factors such as their infectiousness and the severity of infection, and stipulates measures that the government can take to prevent the spread of infection. The Ministry of Health, Labor and Welfare determined that COVID-19 had become a common infectious disease and downgraded COVID-19 to a category five infectious disease on May 8, 2023.

Immediately after the emergence of COVID-19 infection, adverse effects of diabetes mellitus on the outcomes were suspected. Early reports from Wuhan, China, suggested that many patients who died of COVID-19 were complicated with diabetes mellitus [9]. A systematic review and meta-analysis showed that the presence of comorbidities including diabetes mellitus was associated with an increased risk of severity and mortality in patients with COVID-19 [10-14]. These findings were studied before the Omicron epidemic. Few reports examined the impact of diabetes mellitus on the life prognosis of COVID-19 patients after the Omicron epidemic [15]. This study aimed to compare the impact of diabetes mellitus on in-hospital mortality in COVID-19 patients since COVID-19 became a common infectious disease.

## Materials and methods

We performed a retrospective observational study using data from the Osaka General Medical Center (OGMC) and the Osaka COVID-19 Critical Care Center (OC4). OGMC is an advanced critical care center and a disaster-based hospital in Osaka prefecture, Japan. OC4 was a temporary medical facility located within OGMC from December 15, 2020, to March 31, 2023, with an intensive care unit for critical COVID-19. The present study included hospitalized patients treated for COVID-19 at our center between March 1, 2020, and May 7, 2023, before COVID-19 became a category five infectious disease in Japan. Individuals under 18 years old and pregnant women were excluded from the study because their clinical course would potentially differ from the typical prognosis of other adult individuals [16,17]. The patients were all Japanese.

We extracted the following data from the electronic medical records: sex, age, height, body weight, comorbidities (diabetes mellitus, malignant tumor, cardiovascular disease, respiratory disease, liver disease, kidney disease, psychoneurotic disease) [18,19], smoking history, length of hospital stay, cases of a hospital transfer, history of corona vaccination within six months [20], treatments (antiviral drug, neutralizing antibody, steroid hormone, insulin therapy, antibiotics, oxygen administration, intensive treatment, tracheal incubation, extracorporeal membrane oxygenation (ECMO), and blood purification therapy), death during hospitalization, and laboratory findings (plasma glucose level, hemoglobin A1c (HbA1c), white blood cell count, neutrophil count, lymphocyte count, and others).

We detected SARS-CoV-2 using pharyngeal swabs and reverse transcription-polymerase chain reaction (RT-PCR) and diagnosed COVID-19 according to the WHO guidelines [21]. Based on the dates of positive SARS-CoV-2 polymerase chain reaction (PCR) tests in hospitalized patients, we built a histogram classifying the epidemic waves from the first wave to the eighth wave as the previous reports in Japan. After the sixth wave in February 2022, the epidemic mainly affected the Omicron subtype. Therefore, we defined the first wave to the fifth wave as the pre-Omicron epidemic group and the sixth wave to the eighth wave as the post-Omicron epidemic group. We used the American Diabetes Association diabetes mellitus diagnostic criteria when diagnosing diabetes [22]. Patients who received oral hypoglycemic agents or insulins before hospitalization were also considered to have diabetes mellitus. We used the disease registry in the electronic medical records, medical information forms from family physicians, and prescriptions to diagnose complications other than diabetes mellitus.

Since respiratory failure is the most common cause of death in COVID-19 patients, Japan classifies severity based on respiratory symptoms (especially dyspnea) and oxygenation. Measuring SpO_2_ as oxygen saturation, we determined oxygenation status. We classified patients with no pneumonia and SpO_2_ ≥ 96% as mildly ill. We categorized patients with pneumonia and 93% < SpO_2_ < 96% to moderate I. We staged patients with pneumonia and SpO_2_ ≤ 93% requiring oxygenation to moderate II. We sorted patients requiring admission to the intensive care unit or ventilator to severely ill. We performed chest CT scans whenever possible to confirm the presence of pneumonia. When there was a difference in severity between oxygen saturation and clinical status, we classified the patient as severely ill.

We assessed the severity and general condition of COVID-19 patients to determine their overall indication for hospitalization. We admitted COVID-19 patients with moderate disease II or higher and treated them with pharmacologic therapy [23,24]. We also intervened early with oxygen therapy if the patient's respiratory status worsened [25,26]. We empirically administered antimicrobial agents after sputum smear and culture tests if we suspected a complication of bacterial infection based on blood tests and imaging findings. Treatment and testing were determined by routine clinical practice, and we did not implement any interventions designed to be studied.

We divided COVID-19 patients into two groups: pre- and post-Omicron epidemic groups. We then compared risk factors and severity markers of COVID-19 between the groups. Risk factors for severe disease were being male [27], older age (≥65 years), severe obesity (BMI ≥ 30 kg/m^2^), diabetes mellitus, malignant tumor, cardiovascular disease, respiratory disease, liver disease, kidney disease, psychoneurotic disease [13], smoking history, and history of COVID-19 vaccination within six months [20]. Markers of the severity of COVID-19 are lymphocyte count, platelet count, D-dimer, C-reactive protein (CRP), procalcitonin, creatine kinase (CK), aspartate aminotransferase (AST), alanine aminotransferase (ALT), creatinine (Cr), and lactate dehydrogenase (LDH).

The primary endpoint of this study was defined as the in-hospital mortality of COVID-19 patients.

Ethical considerations

The study was approved by the Ethics Committee of the OGMC (approval date: December 22, 2023; approval number: 2023-071).

Statistical methods

We present parametric continuous variables as means ± standard deviation, nonparametric continuous variables as medians (interquartile range), and categorical variables as frequencies (percentage) based on the available data. We used t-tests for comparisons of parametric continuous variables, Mann-Whitney U tests for comparisons of nonparametric continuous variables, and chi-square tests for comparisons of categorical variables. We developed Kaplan-Meier survival curves comparing the two groups, the pre- and post-Omicron epidemic groups. The intergroup difference was evaluated using the Cox proportional hazard regression analysis with death as the objective variable and the post-Omicron epidemic group as the explanatory variable to obtain hazard ratios (HRs) and their 95% confidence intervals (95% CIs). We performed Cox proportional hazard regression analysis with death as the objective variable and risk factors for severe COVID-19 as explanatory variables to obtain HRs and 95% CIs. In addition to each complication, we adjusted for being male, advanced age, severe obesity, smoking history, and COVID-19 vaccination within six months. The covariates entered in the multivariable models were selected based on clinical knowledge and previous reports. As mentioned above, we used COX proportional hazards regression analysis to determine the interaction between diabetes mellitus and the post-Omicron epidemic. We showed the HRs and their 95% CIs for each risk factor for the severity of COVID-19 in forest plots. We performed all statistical analyses using JMP Statistical Software, V.11 (SAS Institute Inc, Cary, NC).

## Results

**Figure 1:**
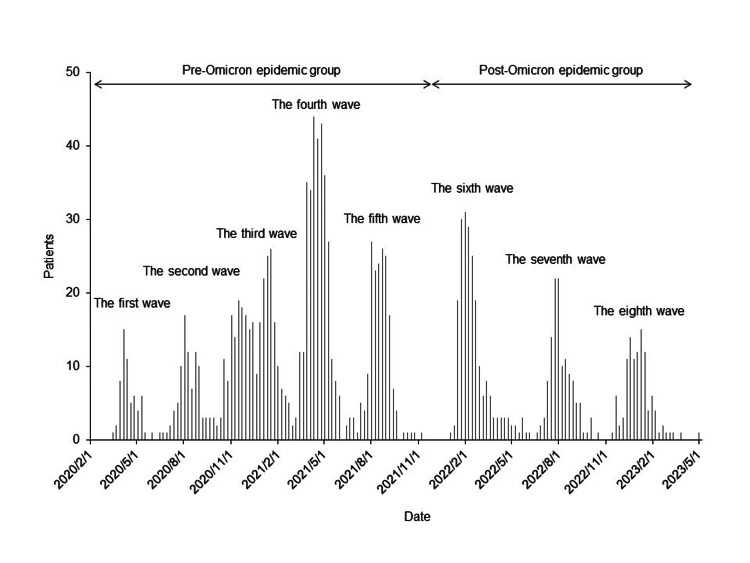
Histogram of SARS-CoV-2 PCR test-positive patients at our center. After the sixth wave in February 2022, the epidemic mainly affected the Omicron subtype. Based on the dates of positive SARS-CoV-2 PCR tests in hospitalized patients, we built a histogram classifying the epidemic waves from the first wave to the eighth wave as the previous reports in Japan. PCR, polymerase chain reaction.

Figure 1 shows a histogram of patients with a positive SARS-CoV-2 PCR test daily at our center. The number of patients was 60 in the first wave, 93 in the second wave, 283 in the third wave, 316 in the fourth wave, 183 in the fifth wave, 211 in the sixth wave, 125 in the seventh wave, 110 in the eighth wave, and 1,381 in total.

Table 1 summarizes the background of COVID-19 patients. We divided patients into two groups, pre- and post-Omicron epidemic, and compared these backgrounds and laboratory findings. The total number of patients was 1,381 (935 in the pre-Omicron epidemic group and 446 in the post-Omicron epidemic group). There were 648 (69.3%) males in the pre-Omicron group and 302 (67.7%) males in the post-Omicron group, respectively, with no significant difference between the two groups (p = 0.5505). The mean age was 67.4 ± 14.5 and 73.0 ± 14.7 years in the pre- and post-Omicron epidemic groups, respectively, with the post-Omicron group being significantly older (p < 0.0001). The mean BMI was 24.4 ± 5.07 kg/m^2^ in the pre-Omicron group and 21.8 ± 5.05 kg/m^2^ in the post-Omicron group, respectively, with the post-Omicron group having a significantly lower BMI (p < 0.0001). The prevalence of diabetes mellitus was 350 (37.4%) in the pre-Omicron epidemic group and 102 (22.9%) in the post-Omicron epidemic group, significantly lower in the post-Omicron group (p < 0.0001). The 452 patients with diabetes mellitus included three with type 1 diabetes mellitus, two with pancreatic diabetes mellitus, five with steroid diabetes mellitus, and the remainder with type 2 diabetes mellitus. The complication rate of diseases that are risk factors for COVID-19 severity, excluding diabetes mellitus and neuropsychiatric disorders, was significantly higher in the post-Omicron epidemic group. The prevalence of kidney disease was 103 (11.0%) in the pre-Omicron epidemic group and 83 (18.6%) in the post-Omicron epidemic group, significantly higher in the post-Omicron group (p < 0.0001). A total of 135 (14.4%) and 113 (25.3%) patients in the pre- and post-Omicron epidemic groups, respectively, had a significantly higher percentage of smoking history in the post-Omicron group (p < 0.001). The proportion of cases transferred from other hospitals was 660 (70.6%) and 169 (37.9%) in the pre- and post-Omicron epidemic groups, respectively, and was significantly higher in the post-Omicron group (p < 0.0001). COVID-19 vaccination history within six months was significantly higher in the post-Omicron epidemic groups, with 38 (4.06%) and 224 (50.2%) in the pre- and post-Omicron groups, respectively (p < 0.0001). The post-Omicron epidemic group had significantly higher rates of antiviral administration, but lower rates of steroid hormones and insulin use (both p < 0.001). The pre- and post-Omicron epidemic groups had statistically insignificant antibiotic use (p = 0.4016). The post-Omicron group had lower rates of oxygenation and tracheal intubation (p < 0.0001). The pre- and post-Omicron epidemic groups had statistically insignificant rates of intensive care (p = 0.1885).

**Table 1:** Clinical characteristics of COVID-19 patients. The analysis divided the epidemic into the pre-Omicron group (the first wave to the fifth wave in Japan) and the post-Omicron group (the sixth wave to the eighth wave in Japan).

	All	Pre-Omicron epidemic group	Post-Omicron epidemic group	
	(n = 1,381)	(n = 935)	(n = 446)	p
Male sex	950 (68.8%)	648 (69.3%)	302 (67.7%)	0.5505
Age, years	69.2 ± 14.8	67.4 ± 14.5	73.0 ± 14.7	<0.0001
Older age, 65 years and over	916 (66.3%)	585 (62.6%)	331 (74.2%)	<0.0001
Height, cm	163 ± 9.96	164 ± 9.77	160 ± 9.99	<0.0001
Body weight, kg	62.6 ± 16.6	65.6 ± 16.3	56.3 ± 15.6	<0.0001
Body mass index (BMI), kg/m^2^	23.6 ± 5.20	24.4 ± 5.07	21.8 ± 5.05	<0.0001
BMI 30kg/m^2^ or more	130 (9.41%)	105 (11.2%)	25 (5.61%)	<0.0001
Comorbidities				
Diabetes mellitus	452 (32.7%)	350 (37.4%)	102 (22.9%)	<0.0001
Malignant tumor	281 (20.3%)	124 (13.3%)	157 (35.2%)	<0.0001
Cardiovascular disease	320 (23.2%)	162 (17.3%)	158 (35.4%)	<0.0001
Respiratory disease	751 (54.4%)	491 (52.5%)	260 (58.3%)	0.0436
Liver disease	123 (8.91%)	66 (7.06%)	57 (12.8%)	0.0005
Kidney disease	186 (13.5%)	103 (11.0%)	83 (18.6%)	0.0001
Psychoneurotic disease	424 (30.7%)	267 (63.0%)	157 (35.2%)	0.0123
Smoking history	248 (18.0%)	135 (14.4%)	113 (25.3%)	<0.0001
Length of hospital stay, days	12 (6-21)	11 (6-21)	12 (6.75-21)	0.9565
Cases of hospital transfer	829 (60.0%)	660 (70.6%)	169 (37.9%)	<0.0001
History of COVID-19 vaccination within 6 months	262 (19.0%)	38 (4.06%)	224 (50.2%)	<0.0001
Treatments				
Antiviral drug	582 (42.1%)	360 (38.5%)	222 (49.8%)	<0.0001
Neutralizing antibody	42 (3.04%)	0 (0%)	42 (9.42%)	-
Steroid hormone	959 (69.4%)	759 (81.2%)	200 (44.8%)	<0.0001
Insulin therapy	529 (38.3%)	431 (46.1%)	98 (22.0%)	<0.0001
Antibiotics	733 (53.1%)	489 (52.3%)	244 (54.7%)	0.4016
Oxygen administration	1,187 (86.0%)	873 (93.4%)	314 (70.4%)	<0.0001
Intensive treatment	769 (55.7%)	532 (56.9%)	237 (53.1%)	0.1885
Tracheal intubation	662 (47.9%)	492 (52.6%)	170 (38.1%)	<0.0001
Extracorporeal membrane oxygenation (ECMO)	34 (2.46%)	31 (3.32%)	3 (0.673%)	0.0030
Blood purification therapy	83 (6.01%)	49 (5.24%)	34 (7.62%)	0.0815
Death during hospitalization	258 (18.7%)	205 (21.9%)	53 (11.9%)	<0.0001

The in-hospital mortality rates for COVID-19, the primary endpoint of this study, are shown in Table 1. A total of 205 (21.9%) patients in the pre-Omicron epidemic group and 53 (11.9%) in the post-Omicron epidemic group died during hospitalization, with the post-Omicron group having a statistically lower in-hospital mortality rate (p < 0.0001).

Table 2 summarizes the laboratory findings of patients with COVID-19. The pre-Omicron epidemic group had a plasma glucose level of 162 ± 77.3 mg/dL, and the post-Omicron epidemic group had 143 ± 78.2 mg/dL; the post-Omicron group had significantly lower plasma glucose levels (p < 0.0001). The pre-Omicron epidemic group had an HbA1c of 6.65 ± 1.19%, and the post-Omicron epidemic group had 6.31 ± 1.23%; the post-Omicron group had a significantly lower HbA1c (p < 0.0001). The lymphocyte count in the pre-Omicron group was 752 ± 523 × 109/L, whereas in the post-Omicron group, it was 808 ± 653 × 109/L, with no significant difference in lymphocyte count between the two groups (p = 0.0923). The pre-Omicron epidemic group had an ALT of 36 (21-60) units/L, and the post-Omicron epidemic group had 21 (13.25-35) units/L, with the post-Omicron epidemic group having a significantly lower ALT (p = 0.0257). The pre-Omicron epidemic group had an LDH of 386 (302-513.5) units/L, and the post-Omicron epidemic group had 271 (210-383.5) units/L, with the post-Omicron epidemic group having a significantly lower LDH (p < 0.0001). The pre-Omicron epidemic group had a serum Cr of 0.75 (0.59-1.01) mg/dL, the post-Omicron epidemic group had a 0.89 (0.63-1.545) mg/dL, and the post-Omicron epidemic group had a significantly higher serum Cr (p < 0.0001).

**Table 2:** Laboratory findings of COVID-19 patients. The analysis divided the epidemic into the pre-Omicron group (the first wave to the fifth wave in Japan) and the post-Omicron group (the sixth wave to the eighth wave in Japan).

		All	Pre-Omicron epidemic group	Post-Omicron epidemic group	
	Reference value	(n = 1,381)	(n = 935)	(n = 446)	p
Plasma glucose, mg/dL	73-109	156 ± 78.0	162 ± 77.3	143 ± 78.2	<0.0001
Hemoglobin A1c, %	4.9-6.0	6.56 ± 1.21	6.65 ± 1.19	6.31 ± 1.23	<0.0001
White blood cell count, ×10^9^/L	3,300-8,600	8,610 ± 4,600	8,850 ± 4,610	8,120 ± 4,540	0.0061
Neutrophil count, ×10^9^/L	1,500-4,700	7,080 ± 4,560	7,460 ± 4,560	6,290 ± 4,480	<0.0001
Lymphocyte count, ×10^9^/L	231-499	770 ± 569	752 ± 523	808 ± 653	0.0923
Red blood cell count, ×10^6^/L	386-492	414 ± 80.3	421 ± 78.8	397 ± 81.2	<0.0001
Hemoglobin, g/dL	11.6-14.8	12.0 ± 2.11	12.3 ± 2.08	11.5 ± 2.06	<0.0001
Platelet count, ×10^9^/L	15.8-34.8	21.0 ± 9.66	21.8 ± 9.73	19.3 ± 9.31	<0.0001
Albumin, g/dL	4.1-5.1	2.61 ± 0.541	2.56 ± 0.501	2.71 ± 0.607	<0.0001
Aspartate aminotransferase (AST), units/L	13-30	36 (25-54)	38 (27-55)	33 (23-51.75)	0.6114
Alanine aminotransferase (ALT), units/L	7-23	31 (18-53)	36 (21-60)	21 (13.25-35)	0.0257
Lactate dehydrogenase (LDH), units/L	124-222	354 (260-475)	386 (302-513.5)	271 (210-383.5)	<0.0001
Creatinine kinase (CK), units/L	41-153	80 (39-183)	71 (36-161)	103.5 (50-271)	0.0552
Serum creatinine (Cr), mg/dL	0.46-0.79	0.79 (0.6-1.1425)	0.75 (0.59-1.01)	0.89 (0.63-1.545)	<0.0001
Estimated glomerular filtration rate, mL/min/1.73m^2^	>90	69.9 ± 37.4	73.9 ± 34.8	61.6 ± 41.1	<0.0001
Blood urea nitrogen (BUN), mg/dL	8-20	22 (16-33)	22 (16-32)	23 (16-39.5)	0.0004
C-reactive protein (CRP), mg/dL	0.00-0.14	6.75 (2.435-12.98)	7 (2.595-12.95)	6.185 (1.98-13.0375)	0.7258
Ferritin, ng/mL	4.63-204	861.5 (391.25-1962.5)	1030.5 (527.5-2288.75)	369.5 (129.5-867)	0.0010
Procalcitonin, ng/mL	<0.05	0.17 (0.08-0.62)	0.16 (0.08-0.42)	0.36 (0.09-1.5425)	0.0022
Sialylated carbohydrate antigen KL-6, units/mL	<500	368 (254-608)	381 (264.75-636/5)	314 (226-456)	0.0051
D-dimer, ng/mL	<0.6	2 (0.9-5.1)	1.9 (0.9-5.3)	2.1 (1-4.975)	0.0888

**Figure 2:**
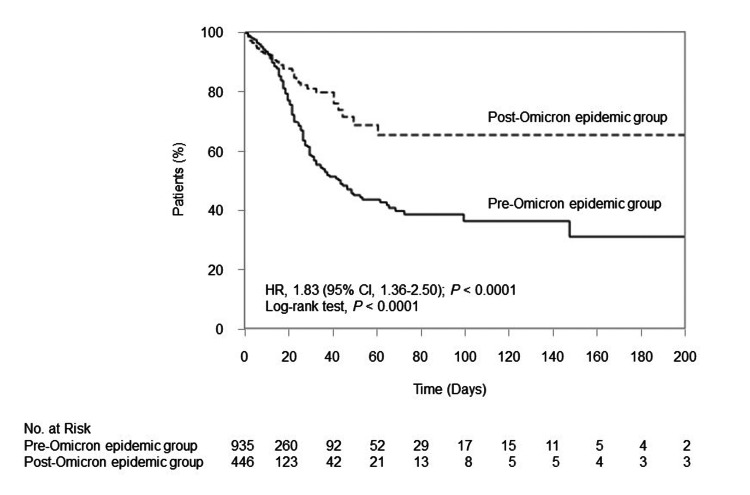
Kaplan-Meier survival curve of COVID-19 patients compared the pre-Omicron epidemic group (a solid line) and the post-Omicron epidemic group (a dashed line) in 935 and 446 patients, respectively. The analysis divided the epidemic into the pre-Omicron group (the first wave to the fifth wave in Japan) and the post-Omicron group (the sixth wave to the eighth wave in Japan). HR, hazard ratio; CI, confidence interval.

Figure 2 shows the Kaplan-Meier survival curve of COVID-19 patients compared to pre- and post-Omicron epidemic. Concerning the primary endpoint, there were fewer death events in the post-Omicron epidemic group than in the pre-Omicron epidemic group (hazard ratio, 1.83; 95% CI, 1.36 to 2.50, p < 0.0001).

Cox proportional hazards regression analysis was used to determine the interaction between diabetes and the post-Omicron epidemic, with p for interaction = 0.2154.

**Figure 3:**
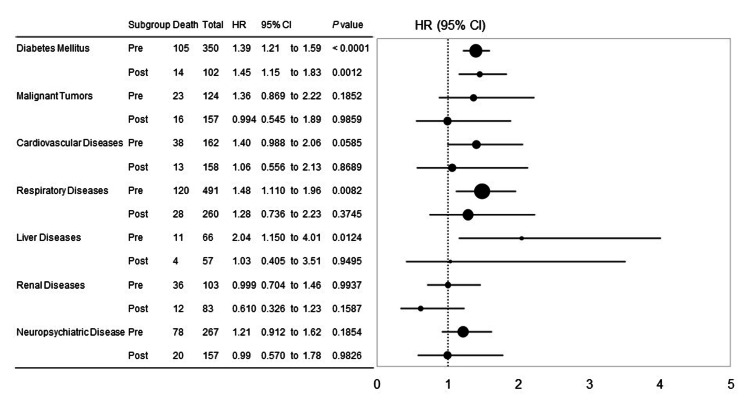
Hazard ratios by risk factor for in-hospital mortality in COVID-19. The analysis was adjusted for factors such as male sex, older age (≥65 years), severe obesity (BMI ≥ 30 kg/m^2^), smoking history, and COVID-19 vaccination within six months, compared to pre- and post-Omicron groups. BMI, body mass index; HR, hazard ratio; CI, confidence interval.

Figure 3 shows hazard ratios for risk factors for death in COVID-19 after adjustment for factors such as older age (>65 years), smoking history, severe obesity (BMI > 30 kg/m^2^), male sex, and COVID-19 vaccination within six months in the pre- and post-Omicron epidemic group. Diabetic patients had higher in-hospital mortality from COVID-19 in both the pre-Omicron epidemic group (HR, 1.39; 95% CI, 1.21 to 1.59, p < 0.0001) and the post-Omicron epidemic group (HR, 1.45; 95% CI, 1.15 to 1.83, p = 0.0012). Only in the pre-Omicron epidemic group, patients with respiratory disease (HR, 1.48; 95% CI, 1.110 to 1.96, p = 0.0082) and liver disease (HR, 2.04; 95% CI, 1.15 to 4.01, p = 0.0124) had a higher COVID-19 in-hospital mortality.

## Discussion

This study was a retrospective observational study conducted at OGMC and OC4 and analyzed in-hospital patients with COVID-19 who were treated from March 1, 2020, to May 7, 2023. The study aimed to investigate the impact of diabetes mellitus on in-hospital mortality of COVID-19 patients in Japan before and after the Omicron epidemic. The duration of each epidemic wave was generally consistent with the trend in Japan. The analysis divided the epidemic into a pre-Omicron group (the first wave to the fifth wave) and a post-Omicron group (the sixth wave to the eighth wave). The results showed a lower in-hospital mortality rate in the group after Omicron administration. The study compared the demographics and test results of the two groups and found differences in age, BMI, diabetes prevalence, comorbidities, smoking history, hospital transfers, COVID-19 vaccination rates, and treatment approaches. Laboratory findings also differed between groups. The pre-Omicron group had higher plasma glucose and HbA1c levels, reflecting the higher prevalence of diabetes. The post-Omicron group also had higher Cr levels, reflecting the higher prevalence of kidney disease. Other risk factors for COVID-19, such as ALT and LDH, were higher in the pre-Omicron group. A decrease in lymphocyte count is known to be a risk factor for the development of severe COVID-19 infection, but this study found no significant difference in lymphocyte counts between the pre-Omicron and post-Omicron epidemic groups. In summary, this study suggests that Omicron variants have a significant impact on the severity and in-hospital mortality of COVID-19, with reduced in-hospital mortality observed in the post-Omicron period. This analysis indicates that diabetes mellitus may hurt in-hospital mortality even in a post-Omicron era.

There was an approval of the new coronavirus mRNA vaccine on February 14, 2021, in Japan. On February 17 of the same year, the vaccination of healthcare workers began as a temporary special vaccination based on the Immunization Law. Subsequently, in addition to the elderly, vaccination has been extended to children aged six months and older. With the widespread use of the COVID-19 vaccine and its replacement by the Omicron sub-strain, the number of patients with COVID-19 infection, a typical viral pneumonia, has decreased significantly [20]. However, the proportion of deaths in Japan among those over 80 years of age is increasing, and exacerbations of underlying diseases, cardiac failure, and aspiration pneumonia should be noted. Old age is the most important risk factor for the severity of COVID-19 [28]. The risk is particularly high for patients with underlying comorbidities such as metabolic diseases (e.g., type 1 and type 2 diabetes, obesity with BMI ≥ 30 kg/m^2^), cardiovascular diseases (e.g., cerebrovascular disease, ischemic heart disease, and cardiomyopathy), and a history of smoking (current and past). Several meta-analyses have shown that men are at higher risk of COVID-19 severity and death than women [10,12,27,29]. However, most findings were made before the Omicron epidemic, and there were few reports after the Omicron epidemic.

Omicron and its sub-strains are much more infectious and transmissible than the pre-Omicron strains but with reduced virulence and a lower proportion of severe cases [7]. Omicron's sub-strains would have similar virulence, infectious immunity, vaccine, and immune escape capabilities. They anticipate that new sub-strains will continue to emerge that have acquired new mutations and are therefore being continuously monitored. The National Institute of Infectious Diseases in Japan has conducted a risk assessment of SARS-CoV-2 variants and classified them as "variants of concern (VOC)," "variants of interest," and "variants under monitoring." As of June 2023, Omicron and its sub and recombinant strains are considered VOCs; COVID-19 infection remains a high-risk health challenge requiring a long-term response. Fourth, the severity levels presented here differ from those of the WHO and the National Institutes of Health, USA [30]. Outside of Japan, the results of this study may not be helpful because of differences in COVID-19 severity classifications and treatment strategies.

This study has several limitations because it is a backward-looking observational study. First, we obtained all data from cohorts of patients admitted to OGMC and OC4. Most of the treated patients were Japanese, and most were severe COVID-19 patients. Thus, risk factors for severe disease may not apply to patients with mild COVID-19. They may also differ among patients belonging to ethnically or geographically diverse populations. Second, the small number of patients in this study may not be sufficiently powerful to reflect the overall complexity of the general population. Therefore, a large prospective cohort study in an ethnically and geographically diverse cohort would be needed to better understand the relevance and importance of diabetes mellitus in COVID-19 disease progression. Third, we could not confirm the type of mutant strain in individual COVID-19 patients in this study. Based on the dates of positive SARS-CoV-2 PCR tests in hospitalized patients, we defined the first wave to the eighth wave and classified the occurrence of COVID-19 mutant strains in Japan as the respective periods.

## Conclusions

The study concluded that omicron mutations had a significant effect on COVID-19 severity and in-hospital mortality, with a reduction in in-hospital mortality observed in the post-Omicron era. Furthermore, this analysis suggests that diabetes mellitus is a noteworthy factor affecting in-hospital mortality even in the post-omicron era when COVID-19 became a common infectious disease.
